# Advances in Hemostatic Hydrogels That Can Adhere to Wet Surfaces

**DOI:** 10.3390/gels9010002

**Published:** 2022-12-22

**Authors:** Wenli Han, Shige Wang

**Affiliations:** School of Materials and Chemistry, The University of Shanghai for Science and Technology, No. 516 Jungong Road, Shanghai 200093, China

**Keywords:** hydrogel, hemostasis mechanism, wet adhesion mechanism, application

## Abstract

Currently, uncontrolled bleeding remains a serious problem in emergency, surgical and battlefield environments. Despite the specific properties of available hemostatic agents, sealants, and adhesives, effective hemostasis under wet and dynamic conditions remains a challenge. In recent years, polymeric hydrogels with excellent hemostatic properties have received much attention because of their adjustable mechanical properties, high porosity, and biocompatibility. In this review, to investigate the role of hydrogels in hemostasis, the mechanisms of hydrogel hemostasis and adhesion are firstly elucidated, the adhesion design strategies of hemostatic hydrogels in wet environments are briefly introduced, and then, based on a comprehensive literature review, the studies and in vivo applications of wet-adhesive hemostatic hydrogels in different environments are summarized, and the improvement directions of such hydrogels in future studies are proposed.

## 1. Introduction

More than 5.8 million people die from severe trauma worldwide each year and approximately 40% of which are caused by uncontrolled bleeding or its consequences [1]. Massive blood loss from incompressible injuries has been reported to cause hemorrhagic shock coagulopathy, multiple organ failure, life-threatening sepsis, and acidosis. Moreover, interventional diagnosis and surgical treatment are prone to hemorrhage or intraluminal hemorrhage, especially in sites close to the heart, parenchymal organs, vital blood vessels, etc. [2]. Therefore, research into hemostatic technologies and materials with superior performance is critical to reducing adverse side effects and even saving lives.

**Figure 1:**
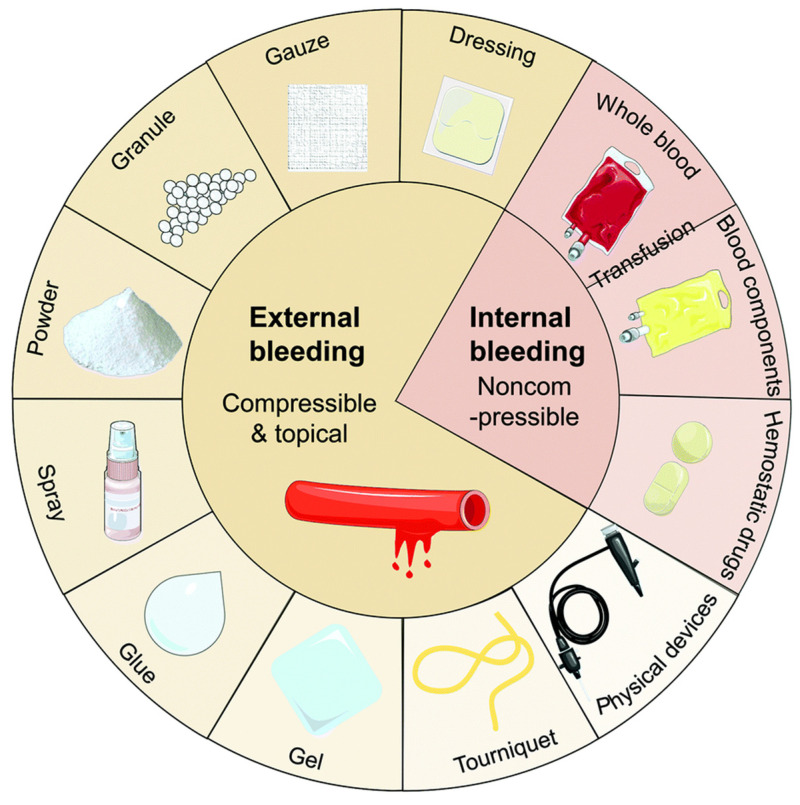
The current methods of hemostasis used for external and internal bleeding management. Figure modified from [11] with permission. Copyright 2020.

Recently, exploring fast and effective methods for controlling bleeding in different application environments has always been an important subject of multidisciplinary research [3]. Sutures and staplers are commonly used to close wounds, however, they have the risk of infection, blood exudation, hyperplasia, and keloid formation [4]. For quick hemostasis in superficial wounds, some conventional hemostatic materials, such as tourniquets [5], hemostatic gauze [6], bandages [7], etc., have been utilized extensively. Nevertheless, common materials struggle to meet the demand for quick and efficient hemostasis in cases of intracavitary hemorrhage or injuries involving essential tissues/organs. Additionally, as gauze and bandages are not biodegradable and can cause secondary injury, delayed healing, and additional discomfort, they need to be removed after hemostasis [8,9]. Therefore, various advanced hemostatic powders, hydrogels, sponges, adhesives, and hemostatic agents [10,11], have been extensively explored (Figure 1).

Adhesives can bind different tissues as well as blood vessels together, whereas hemostatic drugs are known to stop bleeding mechanically or by accelerating the coagulation cascade. Additionally, tissue sealants can stop blood dripping from blood vessels [12]. Nevertheless, some of these products are not universally applicable and have obvious disadvantages. For example, fibrinogen and thrombin-based injection solutions (fibrin sealants) may be washed away by the bloodstream and cannot be used effectively in severe or emergency arterial bleeding [13,14]. As emerging medical adhesives, cyanoacrylates may cause allergic reactions, and their rapid curing process is exothermic [15]. Furthermore, such products have not been used inside the body due to concerns about the potential toxicity of the degradation products. As a consequence, designing an ideal hemostasis technique for effective hemostasis in complicated clinical situations remains challenging. 

Hydrogel-based biomaterials show many advantages over traditional hemostasis methods [16,17]. A hydrogel is a 3D cross-linked hydrophilic polymer with a structure similar to that of the natural extracellular matrix (ECM) [18]. They are widely used in biomedicine, and due to their injectability and fluidity, hydrogels are crucial for achieving quick and lasting hemostasis for a variety of irregular wounds and intraluminal injuries [19]. In addition, the hydrogel-based biomaterial is safe for use in vivo due to its exceptional biocompatibility and biodegradability [20,21]. An ideal polymer hydrogel for hemostatic applications should have the following properties: (i) the hydrogel should be able to effectively promote wound healing and immediately block the bleeding, [22] (ii) the hemostatic hydrogel should have superior mechanical qualities that enable it to quickly adhere and completely seal the damage, especially in humid and dynamic settings, and [23] (iii) the hydrogels must have quick coagulation to prevent the migration of hemostatic hydrogels from the bleeding site [24]. 

High pressures and blood flow rates may remove or destroy general hydrogels due to their limited underwater adhesion and weak adhesion mechanisms [25]. Therefore, it is crucial to enable hemostatic hydrogels to adhere to tissues and maintain their integrity in humid environments. Moreover, an ideal wet-adhesive hydrogel should completely cover the wound area, minimize surgical scope or complications, carry therapeutic cells and drugs, and release them to the wound site [26]. This review introduces the properties of hemostatic hydrogels that can adhere to wet surfaces, briefly discusses the basic principles of hemostasis, and then focuses on the adhesion mechanism of hydrogels on wet surfaces. This review is anticipated to promote further research progress on wet viscous hemostatic hydrogel and create more possibilities for rapid and effective emergency hemostasis.

## 2. Properties of Wet Adhesive Hemostatic Hydrogel

The complicated dynamic equilibrium environment of the human body should be adapted when hydrogels are employed to stop bleeding. The ideal adhesive hemostatic hydrogel should possess the properties of strong wetting adhesion, hemostasis, antibacterial, and biocompatibility.

### 2.1. Hemostatic Properties

**Figure 2:**
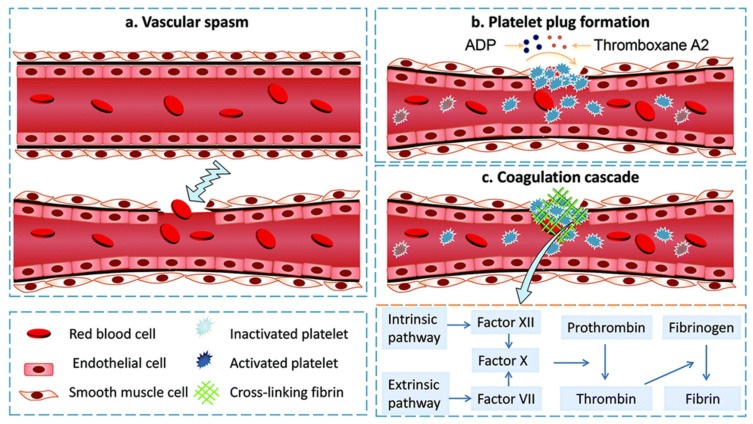
Schematic diagram of the hemostatic process: (**a**) vascular spasm. Immediately after injury, blood vessels are stimulated to constrict to reduce blood loss; (**b**) platelet plug formation. Once platelets are activated, the shape changes, releasing serotonin, ADP, and thromboxane A2, etc., causing more platelets to concentrate at the site of injury to form a thrombus; (**c**) coagulation cascade. Endogenous and exogenous coagulation pathways are activated, involving the interaction of factor (XII) and coagulation factor VII in the blood, with the two pathways eventually converging into a common pathway that activates factor X, which further converts prothrombinogen to thrombin. The generated thrombin activates Factor XIII, which promotes the conversion of fibrinogen into fibrin chains for hemostasis. Figures modified from [11] with permission. Copyright 2020.

Hemostasis is the first stage of wound healing, starting with wound formation and ending with thrombosis. The physiological mechanism of human hemostasis is a complex dynamic process consisting of three processes: vasoconstriction, formation of platelet embolism, and coagulation (Figure 2). Vasoconstriction is a transient reflex contraction that is the first step in preventing blood loss during injury (Figure 2a). Thromboembolism is the second critical stage of hemostasis, involving platelet adhesion, activation, and aggregation into embolization. During the damage, platelets bind to von Willebrand factor (VWF) aggregates via their GP1b receptor and directly adhere to exposed subendothelial collagen via two receptors (glycoprotein VI (GP VI) and integrin α2β1) [27]. This adhesion triggers an activation process of platelets, which leads to a change in shape and the release of particles rich in substances such as serotonin, thromboxane A2 (TXA2), and adenosine diphosphate (ADP). Because they alter the conformation of GPIIb/IIIa, a receptor on the surface of platelets that enables platelets to bind to fibrinogen and induce platelets to aggregate into the walls of damaged blood arteries (Figure 2b). Primary hemostasis is a brief stage of hemostasis that finally results in the creation of a first embolism. The extrinsic and intrinsic pathways are two separate processes that take place during the second stage of hemostasis [28]. The extrinsic pathway, which starts when a blood artery is damaged, involves the interaction of tissue factor (TF) that is exposed to the blood and coagulation factor VII (FVII). When factor XII comes into contact with a foreign substance having a negatively charged surface, intrinsic pathways are activated. The activation of factor X, an enzyme that assists in the conversion of prothrombin to thrombin (thrombin transforms fibrinogen to fibrin monomers), is the result of these two processes (common pathway). Finally, fibrin monomers self-polymerize into fibrils, which laterally aggregate to create fibers and establish a hydrogel (Figure 2c). The interfiber and interfiber crosslinking caused by factor XIIIa stabilizes the fibrin gel and enhances clot stiffness.

According to cell-based models of hemostasis, the coagulation process has three overlapping stages: initiation, amplification, and propagation. When TF-bearing cells are exposed to flowing blood after vascular damage, the initiation phase begins, which results in the generation of activated factors IX and X as well as thrombin. Such thrombin serves several important roles during the amplification stage. Along with activating platelets, it also causes the cofactors V and VIII to become Va and VIIIa on the surface of the activated platelets. Thrombin activates factor XI on the surface of the platelet during this stage [8]. The proliferation that takes place on the surface of activated platelets during the last stage of this cellular model and eventually results in a burst of thrombin that is sufficient to create the fibrin mesh. Hydrogels can facilitate the hemostasis process on wet surfaces through three typical approaches: i) direct or indirect participation in the coagulation system to activate the physiological hemostatic process; ii) enrichment of the wound site with coagulation components (e.g., polymeric polysaccharides, inorganic zeolite clay, etc.) by physical and chemical means; iii) physical closure of blood vessels using the strong adhesion of the material to the tissue.

### 2.2. Adhesion Properties

#### 2.2.1. Four Theories of Bio-Adhesion

**Figure 3:**
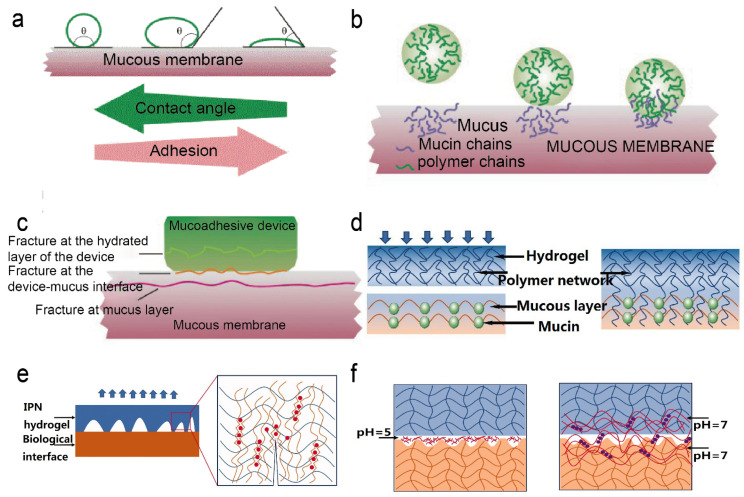
(**a**) Wetting theory. (**b**) Diffusion theory. (**c**) Fracture theory. Figures modified from [35] with permission. (**d**) Mechanical theory. (**e**) The principle of double network hydrogel enhances adhesion. The blue network represents polyacrylamide, the orange network represents alginate, and the red dots represent calcium ions. (**f**) Principle of topological binding. The chitosan chains are dissolved in a solution at pH = 5. The chitosan chains diffuse into the gel at pH = 7, using hydrogen bonds to form a new network. Figures modified from [36] with permission.

There are four typical bio-adhesion theories, including wetting, mechanical, diffusion, and fracture theories [29] (Figure 3). Wetting is a phenomenon caused by liquid diffusion along a solid surface. Surface tension, capillary force, Van der Waals force, etc., are the basic causes of wet adhesion [30] (Figure 3a). Wet adhesion is an approach in which hydrogels can enhance adherence. According to the wetting hypothesis, capillary actions are brought on by curved water surfaces and tight contact between the interfaces, which results in short-distance interactions [31]. The heterogeneity of the hydrogel is a key element in determining its adhesiveness. Hydrogel adhesions to hydrophobic surfaces are poor in the air, yet they may cling to hydrophilic surfaces quite effectively. This is because water will moisten the surface and create a water meniscus at the border of the contact region. As a consequence, in addition to molecular interactions, capillary adhesions also exist at the interface of the two adhesives [32]. The groups on the polymer can interact with the interface, however, when a hydrogel is submerged in water, pressure is usually needed to remove free water. The interface adhesion is related to the surface energy, therefore, appropriate surface energy is necessary to afford firm adhesion [33]. Inspired by the structures of tree frog toes, Nguyen et al. evaluated the wet stickiness of the contact interface under various circumstances [34]. The findings demonstrated that, in comparison to the surface of the non-patterned plate, the micro pattern cushion’s surface boosted the interface adhesion in a wet environment. The effect of contact shape and substrate morphology on adhesion was investigated by Liu et al. [30]. The findings demonstrated that the interstitial liquid’s contact geometry, and capillary density are strongly connected to the wet adhesion, and the ratio of capillary and adhesive forces is key to elucidating the mechanism of wet adhesion.

On the other hand, a rough interface, in accordance with mechanical theory, increases the surface area that can make contact as well as the viscoelastic and plastic energy dissipation during joint breakage (Figure 3d) [35]. The first bonding hypothesis, known as mechanical interlocking (or mechanical interlocking), was proposed by MAC Bain and Hopkins in 1925 [37] and referred to the interlocking of the adhesive with the microscopic rough surface of the bonded object. Traditional alveolar bone filling methods achieve adhesion between the alveolar bone and the pretreated tooth surface, which is facilitated by mechanical interlocking [38]. Inspired by this phenomenon, bonding and adhesive can be achieved by filling the hydrogel in the pores of the substance. By utilizing the catechol-mediated synergized adhesion and interlocking in the pores of diatom silica, Lee et al. prepared a diatomaceous earth/polysaccharide elastic hydrogel, which showed good biocompatibility and can be strongly adhered to the skin [39]. In a separate study, sodium alginate and acrylamide were employed as raw ingredients by Yuan et al. to create a mechanically interlocked double-net hydrogel with enhanced adhesive capabilities and mechanical properties [40]. Such a hydrogel was competent in blocking the perforation and encouraging the healing of tissues.

In addition, the adhesion of the hydrogel to the mucosal layer can be explained by diffusion theory. For instance, certain bioadhesive polymers may dissolve in mucus mucin, and flexible polymer chains can physically entangle with mucin chains to provide an advantageous adhesion effect (Figure 3b) [35]. According to the theory of diffusion, adhesion is produced by the reciprocal diffusion of molecules between the substrate and the adhesive, therefore, macromolecular chains require sufficient solubility and flexibility for mutual diffusion. Due to the presence of an interpenetrating network structure, the hydrogel can create interdiffusion among polymers to increase its adhesion qualities. Feng et al. prepared silk fibroin/konjac glucomannan sponges with an interpenetrating network for wound dressing, which showed high biocompatibility for cell adhesion and proliferation [41]. In another study, Vorwald et al. created fibrin-alginate interpenetrating network hydrogels, which combined the excellent adhesion and stimulatory characteristics of fibrin with the adaptable mechanical properties of alginate for cellular orientation and distribution by combining [42]. The fracture theory was typically used to determine the maximal tensile stress. The toughness of the stiff polymer without flexible chains may also be calculated using this method (Figure 3c).

#### 2.2.2. Influence Factors of Wet Surface Adhesion

**Figure 4:**
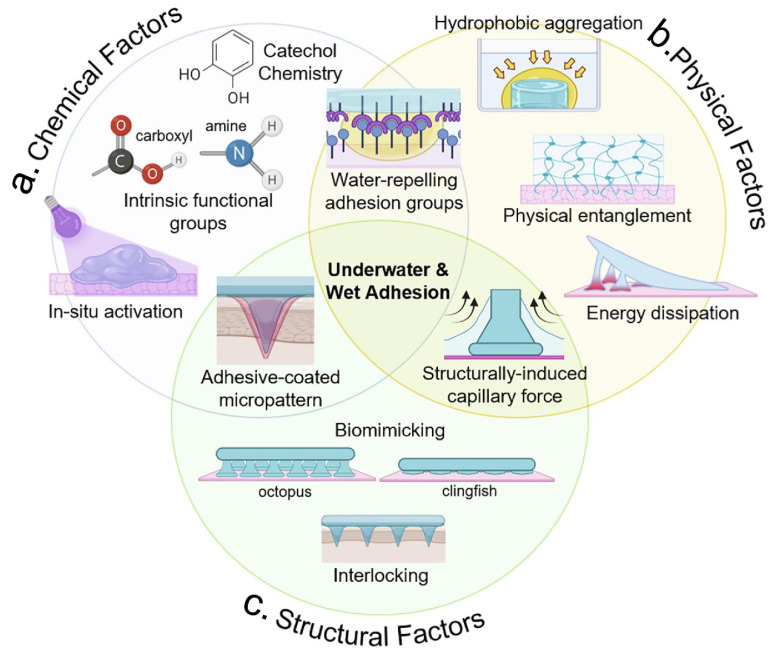
The practical application of hydrogels in wet adhesion involves complex interactions that can be divided into three main categories: chemical, physical, and bionic structural factors. (**a**) Chemical factors, such as catechol chemistry, in situ activation, and reactions of intrinsic groups of the material. (**b**) Physical factors, such as hydrophobic aggregation, physical entanglement, and energy dissipation. (**c**) Biomimetic structural factors, such as micro-patterning and capillary forces. Figure modified from [43] with permission.

In practice, the adhesion of hydrogels involves a complex interaction of chemical, physical and structural factors (Figure 4). Therefore, understanding these factors will help us to design the wet adhesion strategy for a given application. In this section, the chemical, physical, and structural factors that influent the wet surface adhesion of hydrogels are briefly summarized (Table 1).

(1) Typical Chemical Bond

The adhesion capacity of hydrogels is usually related to the formation of ionic, covalent, and metal coordination bonds between the hydrogels and tissue [44]. Covalent bonding, in which functional groups chemically react with groups on the target surface, is the primary way to enhance adhesion. Covalent bonds have higher underwater adhesion strength than intermolecular forces because they require sharing of electrons. By creating covalent bonds, hydrogels exhibit underwater adhesion and cohesion, making them attractive to substrates containing reactive functional groups [45]. When the hydrogel is used underwater, the presence of chemical or physical crosslinks prevents it from over-swelling and impairs adhesion [46]. However, covalent-based underwater bonding usually involves surface modification or is dependent on the specific adhered surface [47]. Catechol chemistry is one of the most frequently used interactions among the many functional groups. Studies have uncovered facile methods to synthesize catechol polymers and molecules that can be grafted and coated multifunctionally into other materials [48]. Due to their inherent good biocompatibility, catechols are widely used for tissue adhesion in humid environments [49] (Figure 4a). Moreover, the groups in catechol hydrogels (containing ortho-quinone groups) can establish covalent bonds with the nucleophiles on the surface of the tissue and thus adhere to the membranes of biological tissues [50]. Zhou et al. spliced the catechol moiety of dopamine (DA) in the polymer chain of hyaluronic acid hydrogels, which established covalent bonds with nucleophiles (amines, thiols, and hydroxyl groups) and thus adhered to the wet biological tissue [51]. In another study, Ma et al. prepared in situ photo-responsive chitosan (CS) hydrogels based on the imine cross-linking method. After exposing the hydrogels to UV light, *o*-nitrophenyl was converted to the *o*-nitrobenzaldehyde group. This group was further cross-linked with the amino group on the tissue surface, thus forming a covalent bond that allows the wet tissue adhesion [52].

Covalent connections can be further classified into permanent and dynamic covalent bonds in physiological contexts, which have significant advantages in the application of hydrogel bio-adhesion. Permanent covalent bonds can be carbon–sulfur, carbon–carbon junctions, carbon–nitrogen, and silicon–oxygen bonds. Permanent covalent bonds are stable, hard, and irreversible. By irreversibly rupturing covalent or neighboring bonds, these bioadhesive hydrogels can be separated. Adhesives with permanent covalent typically adhered to the surface by reacting with other functional groups. Dynamic covalent bonds (DCBs) can reversibly break and form covalent bonds under specific conditions. DCBs can be formed by the linkage of disulfides, imides, hydrazides, phenylboronic acids, and Diels–Alder reactions. The use of dynamic covalent bonding, combined with the favorable properties of chemical and physical adhesion, allows the design of robust and reversible bioadhesive hydrogel dressings. During the adhesion process, DCBs can assemble a hydrogel network with self-healing capabilities between two adherent gel networks or interfaces. Such gels are widely used for wound closure and wound hemostasis. For example, Yang et al. developed an injectable mussel mucoadhesive self-healing hydrogel with good bioadhesive and hemostatic properties based on C-N single bonds and C-N double bonds [53]. Compared to conventional covalently linked hydrogels, hydrogels containing DCBs exhibited significant self-healing ability and good cytocompatibility. Using aminoglycoside, aldehyde-based hyaluronic acid, and adipic dihydrazide grafted hyaluronic acid as raw materials, Li et al. created the dynamic covalently cross-linked hydrogels. Such hydrogels were formed based on the imine and hydrazone cross-linking and showed strong and long-lasting antimicrobial properties, good biocompatibility, and self-healing ability [54].

(2) Hydrogen Bond

**Figure 5:**
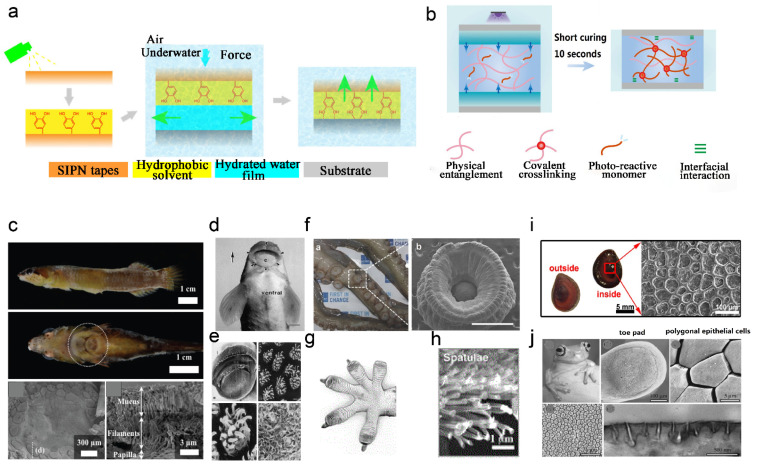
(**a**) Exclusion of interfacial water to enhance adhesion strategy. Figure modified from [58] with permission. (**b**) Absorption of interfacial water and rapid light curing strategies. Figure modified from [62] with permission. (**c**) Clingfish photos and SEM images of adherent disc surface morphology. Reprinted (adapted) with permission from [80]. Copyright {2022} American Chemical Society. (**d**) Teleost photographs and (**e**) SEM images of mouth surface morphology. Figure modified from [81] with permission. (**f**) Photographs(a) of octopus tentacles and SEM images(b) of its suckers (scale bar: 1 mm). Figure modified from [82] with permission. (**g**) Ventral diagram of the gecko foot. (**h**) SEM diagram of foot setae morphology. Figure modified from [83] with permission. (**i**) Photographs of the gill cover of the snail and SEM images of the surface morphology. Reprinted (adapted) with permission from [84]. Copyright {2020} American Chemical Society. (**j**) Photographs of tree frogs and SEM and TEM images of the surface morphology. Figure modified from [85] with permission.

A hydrogen bond is created by the dipole–dipole attraction of two electronegative atoms. The hydrogen bond is an important driving force for hydrogel formation. For example, a novel pH-sensitive hydrogel was prepared by compositing konjac glucomannan, dopamine hydrochloride, L-cysteine hydrochloride, and epigallocatechin acid esters. Hydrogen bonds and catechol-mediated coordination were responsible for the hydrogel formation. This hydrogel possessed injectability and adhesion properties for potential use in drug delivery and release controllability [55]. In another study, Zhang et al. designed the catechol-modified polylysine/polyacrylamide hydrogel. The numerous hydrogen bonds rendered the hydrogel’s strong adhesion, high compressive strength, and effective hemostasis, and can be used to seal bleeding sites [56]. Typically, there existed a counterpart between the hydrogel-wet surface hydrogen bonds and water molecules–induced hydrogen bonds. To enhance the wet surface adhesion of hydrogel, the water molecules-induced hydrogen bonds should be suppressed. A typical way to inhibit hydrogen bonding caused by water molecules is to repel or absorb water molecules from the surface (Figure 5a,b). The water film formed by these water molecules is considered to be the hydration layer. At the molecular level, the hydration layer at the hydrogel-attachment interface can be resisted by hydrophobic interactions. Therefore, hydrophobic solvents and hydrophobic monomers are usually used in hydrogels in order to improve wet adhesion [45,57]. Spraying the hydrophobic solvent on the surface of the hydrogel can also form a thin hydrophobic layer with a small contact angle with water [58]. When external pressure is applied to the hydrogel, the hydration layer can be broken and discharged from the adhered surface. Similarly, some hydrogels contain hydrophobic monomers (e.g., with long carbon chains or aromatic ring structures) that can repel water molecules and stick firmly thereto [59,60].

In contrast, the method of absorbing the hydration layer is simple than repelling the hydration layer. The absorption of the hydration layer is based on the fact that hydrogels contain highly hygroscopic materials [61]. For example, Wang et al. prepared an in situ photocuring hygroscopic hydrogel adhesive by using polyvinylpyrrolidone (PVP, as a hygroscopic polymer), acrylic acid, crosslinker, and photoinitiator [62]. Cong et al. designed an anthracene-based polyethyleneimine underwater adhesion hydrogel, and the high water absorption of polyethyleneimine can promote the absorption of interfacial water molecules and enable the hydrogel to adhere firmly to other substances [63].

(3) Van der Waals Force

Environmental stimuli can easily disrupt some of the adhesions generated based on chemical reactions. By contrast, less reactive physical factors can be used to durably strengthen wet adhesion. Van der Waals forces, electrostatic forces, hydrogen bonding, and hydrophobic interactions are representative examples of non-covalent interactions between interfaces, which affords physical adhesion. Van der Waals force is a weak force, especially in water, therefore, it is not the main driver of the adhesion of hydrogels and other substances. However, van der Waals forces can be aggregated into large volumes of material to provide strong adhesion. For example, geckos can stay and crawl normally on vertical walls due to the aggregated van der Waals forces generated by the contact of their feet with the walls [64]. Yi et al. created a bio-inspired wet hydrogel adhesive using polyethylene glycol hydrogel as a starting material by exploiting the water absorption properties, capillary forces, and van der Waals forces of hydrogels [65]. The mixture has excellent biological application potential and exhibited extraordinary reversible adherence to dry, wet, and submerged substrates. In another study, Sato et al. found that the Van der Waals forces are responsible for the adhesion of submicron silica particles to the surfaces of poly(acrylamide) and poly(dimethyl-acrylamide) hydrogels [66].

(4) Electrostatic Interactions

Adding electrostatic interactions to hydrogels can improve biological wet adherence. Through electrostatic interactions, hydrogels can firmly adhere to the surface of different substances [67]. To create hydrogels with strong adhesion, Huang et al. prepared hybrid hydrogels with ionic and hydrophobic cross-linked networks, with strong adhesion and high toughness properties attributed to the synergistic effect of electrostatic interactions and hydrophobic junctions [68]. Tian et al. designed a strong and stable adhesive hydrogel with synergistic hydrophobic interactions and dynamic electrostatic forces using 2-acrylamido-2-methylpropanesulfonic acid, gelatin, CS, ethyl 2-methoxyacrylate, and acrylic acid as raw materials. The hydrogel could adhere stably and firmly to various tissue surfaces [69]. Song et al. prepared a multifunctional physical hydrogel adhesive using catechol-modified CS and polyvinyl alcohol as raw materials. Dynamic hydrogen bonding and electrostatic interactions improved the persistence and reproducibility of this hydrogel adhesive [70].

(5) Metal Coordination Bond and Ionic Bond

The metal coordination bond and ionic bond also affect the wet surface adhesion. A specific type of covalent link known as a metal coordination bond typically has a stronger binding than a hydrogen bond [71]. The coordination bond is reversible, which gives the hydrogel reversible underwater adhesion characteristics in addition to maintaining the stability of the covalent bond [72]. An ionic bond contains two opposite-charged ions, which are also stronger than hydrogen bonds [73]. In order to create strong and durable ampholyte hydrogels, Huang et al. created a synthetic polyamphiphilic electrolyte hydrogel using the complementary interaction of ionic and metallic coordination bonds [74]. Using acrylamide, sodium alginate, and acrylic acid as raw materials, Liang et al. created an ultra-strong and tough hydrogel with excellent mechanical properties, adhesion, high hardness, toughness, fatigue resistance, and salt resistance. This hydrogel formation was based on the establishment of strong ionic and weak hydrogen bonds [75].

**Table 1:** Influencing factors of wet surface adhesion.

Influence Factors	Hydrogels	Adhesion Mechanism
Permanent and dynamic covalent bonds	Hyaluronic acid and dopamine polymerized hydrogel [51] In situ photo-responsive chitosan (CS) hydrogel [52]	Dopamine catechol chemical adhesion After exposure to UV light, o-nitrobenzene is converted to o-nitrobenzaldehyde, which is further cross-linked with amino groups on the tissue surface.
Hydrogen bond	Konjac glucomannan, dopamine hydrochloride, L-cysteine hydrochloride, and epigallocatechuic acid ester pH-responsive hydrogels [55] Catechol-modified poly(lysine)/poly(acrylamide) hydrogels [56] Anthracene-based polyethyleneimine underwater adhesion hydrogel [63]	Hydrogen bonding and catechol chemical adhesion Strong adhesion of hydrogel due to large amount of hydrogen bonding The high water absorption of polyethyleneimine promotes the absorption of interfacial water molecules, allowing the hydrogel to adhere firmly to other substances
Van der Waals force	Polyethylene glycol hydrogel [65]	Water absorption properties, capillary and van der Waals forces
Electrostatic interactions	2-Acrylamido-2-methylpropanesulfonic acid, gelatin, CS, ethyl 2-methoxyacrylate, and acrylic acid bonded hydrogels [69] Catechol-modified CS and polyvinyl alcohol were used as raw materials to prepare physical hydrogels [70]	Synergistic hydrophobic and electrostatic interactions Dynamic hydrogen bonding and electrostatic interactions
Metal coordination bond and ionic bond	Acrylamide, sodium alginate, and acrylic acid super tough hydrogel [75]	Strong ionic and weak hydrogen bonding interactions

(6) Biomimemtic Strategies

Research interest in the adhesion abilities of mussels, tree frogs, geckos, snails, teleosts, clingfish, and octopuses has led to the development of biomimetic hydrogel adhesives (Figure 5c–j). Biomimetic adhesions mirror the surface interactions caused by the topological structure of naturally adhesive creatures and are less sensitive to environmental cues. The adhesion of geckos and octopuses to wet surfaces primarily originated from the concurrence of physical forces like negative pressure, capillary force, and mechanical interlocking [76]. The sucker patterns, which exhibited a strong capillary force, were mimicked to elevate the wet adhesion content of polyethylene glycol (PEG) hydrogel [65,77] (Figure 4c). The hydrogel micropillars expanded when placed on a moist surface, which created a capillary force around the suction cups and moved the micropatterns closer to the substrate for adherence. In another study, a PDA hydrogel was tailored to present the micro-channels and mimic the dynamics of the suction cups of geckos and octopuses. Such a PDA hydrogel was able to contract upon heating and expand when cooled, during which a negative pressure was created to suck the surface water into chambers to enable wet surface adherence [78]. Recently, an endoparasites-inspired hydrogel-forming double-layered adhesive microneedle patch, which is composed of a non-swellable silk fibroin-based core and swellable mussel adhesive protein-based shell, was proposed. The double-layered adhesive microneedle patch showed enhanced tissue insertion capability and superior wound-sealing capacity for wet and/or dynamic external and internal tissues [79]. The cohesion (mechanical properties) of the hydrogel is also closely related to the wet surface adhesion strength, as good mechanical properties support the deformation of the hydrogel without breaking. The duration of adhesion is equally important for practical purposes, as biomedical applications require that the hydrogel remains adherent to the implant site until effective hemostasis, tissue regeneration, or therapeutic delivery is complete. For example, the catechol-mediated wet surface adhesion was prone to disrupt by natural oxidation. Accordingly, protecting it from oxidation is the main strategy to enhance wet adhesion [46]. 

## 3. Application of Wet Adhesion Hemostatic Hydrogels

In this section, the applications of wet adhesion hydrogels for the hemostasis of skin, heart, liver, and other kinds of bleeding were summarized.

### 3.1. Skin

**Figure 6:**
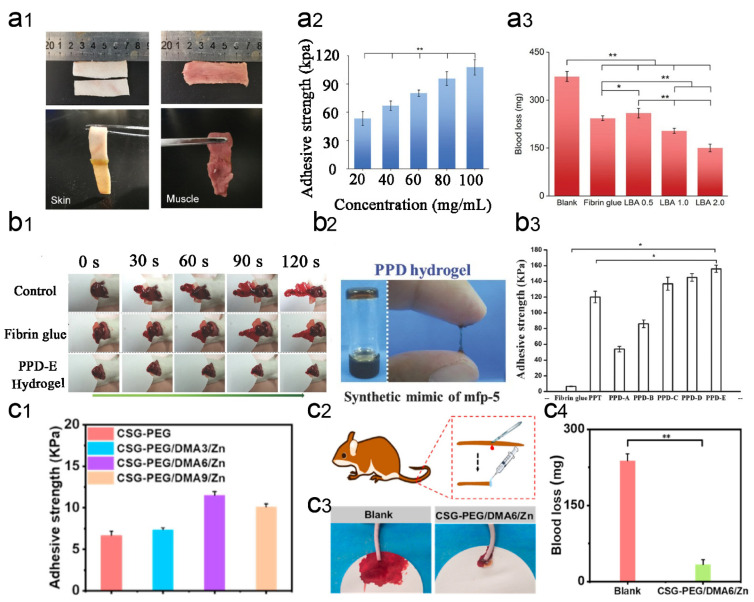
Application of wet-adhesive hemostatic hydrogel in skin wounds. (**a**) Adhesive properties and hemostatic effect of LBA hydrogel. Figure modified from [52] with permission. (**a1**) Photographs of tissue adhesion using LBA in various tissues (pig skin and muscle). (**a2**) Tissue adhesion strength of LBA 1.0 with different precursor solution concentrations. (**a3**) Blood loss during hemostasis with LBA in a rat hepatic hemorrhage model. (**b**) Wet adhesion properties and hemostatic effect of PDD hydrogel. Figure modified from [88] with permission. (**b1**) Total view of livers of bleeding mice treated and untreated with PPD hydrogel, and fibrin glue every 30 seconds for 2 minutes. (**b2**) PPD hydrogels are prepared through an HRP cross-linking reaction. (**b3**) Adhesion strength of PPD hydrogel to porcine tissues. Adhesion of p-nitrophenylchloroformate/PEG/TA hydrogel (PPT) and fibrin glue was used as a control (n = 5, * *p* < 0.05). (**c**) Adhesion and hemostatic properties of CSG-PEG/DMA/Zn hydrogels. Figure modified from [89] with permission. (**c1**) Adhesion strength of hydrogel. (**c2**) Schematic diagram of the mouse tail amputation model. (**c3**) Photographs of blood stains in a mouse tail amputation model. (**c4**) Quantitative results of blood loss in a mouse tail amputation model (n = 4, * *p* < 0.05, ** *p* < 0.01.).

The skin is the largest multi-layered organ in the human body, consisting of the epidermis and dermis [86]. When the entire epidermis is severely injured, the skin loses its basic defense layer, and microbial infection at the wound site can slow the healing process [87]. Unlike other phospholipid-containing biofilms, the skin surface contains amino, carboxyl, and hydroxyl groups that contribute to the wet adhesion of the hydrogel. The Schiff base reaction can be used to cross-link the aldehyde group in the hydrogel with the amino group on the skin tissue to achieve stronger tissue adhesion. For example, Ma et al. prepared a liquid bandage (LBA), which is an in situ imine cross-linked photoactive CS hydrogel (NB-CMC/CMC hydrogel) [52]. NB-CMC was synthesized by modifying o-nitrobenzyl alcohol (NB) with water-soluble carboxymethyl chitosan (CMC). Under UV irradiation, o-nitrobenzene was converted to an o-nitrosobenzaldehyde moiety and cross-linked with amino groups on the tissue surface, resulting in excellent tissue adhesion. The hemostatic and antimicrobial properties of LBA were correlated with the mass ratio of NB-CMC/CMC. LBA exhibited acceptable biocompatibility and biodegradability, can effectively control bleeding, produce strong tissue adhesion, avoid bacterial infection, and accelerate wound healing (Figure 6a). Other molecules, such as sulfhydryl groups, long-chain alkyl groups, or DA-like compounds, can also be added to the hydrogel to enhance wet surface adhesion. In a study inspired by mussel mucin, a DA modified ε-poly-lysine-polyethylene glycol-based hydrogel (PPD hydrogel) wound dressing was developed in situ using horseradish peroxidase (H_2_O_2_/HRP) cross-linking method. It was shown that the PPD hydrogel had good wet tissue adhesion properties and exhibited excellent hemostatic effects to accelerate skin wound repair [88] (Figure 6b). In other studies, Lu and co-workers prepared several skin adhesive hydrogels based on the adhesion ability of DA [89,90]. These hydrogels can increase shear viscosity by an average of 10–30 kPa. 

In addition, metal ions (e.g. zinc ions, silver ions, calcium ions, etc.) can introduce additional functionality to the hydrogel [91]. For example, Wang et al. developed an injectable and in situ photo-crosslinked hybrid hemostatic hydrogel by combining pectin methacrylate (PECMA) and methacrylate-based gelatin (GelMA). It was shown that the PECMA/GelMA hydrogel has good cytocompatibility and synergizes the hemostatic properties of calcium ions on PECMA, amine residues on GelMA, and a highly porous network to achieve rapid blood absorption and coagulation. An in vitro porcine skin bleeding model confirmed that the hydrogel could be injected directly into the wound and rapidly photo-crosslinked, and reduce the coagulation time by 39%. Importantly, the hydrogel can be easily removed to prevent secondary injury to the wound [92,93]. In another study, Yang et al. developed a photo-crosslinked multifunctional antibacterial and antioxidant hemostatic hydrogel dressing. It contained polyethylene glycol monomethyl ether modified glycidyl methacrylate functionalized CS (CSG-PEG), methacrylamide dopamine (DMA), and zinc ions. In a mouse model with intact skin defects infected with methicillin-resistant Staphylococcus aureus, CSG-PEG/DMA/Zn hydrogel not only adhered well to the wound surface but also showed better hemostasis and promoted wound healing of infected skin tissue defects than the commercially available Tegaderm^TM^ film [94] (Figure 6c). For faster clotting to prevent chronic inflammatory episodes, Chen et al. prepared a novel hemostatic hydrogel by cross-linking inorganic polyphosphate (PolyP) conjugated with polyaspartic hydrazide (PAHP) and PEO_90_ dialdehyde (PEO_90_ DA) [95]. The dynamic nature of the acyl-hydrazone bond allowed the hydrogel to self-repair when damaged by external forces. The hydrogel simultaneously exhibited excellent tissue adhesion, biocompatibility, antimicrobial activity, and hemostatic efficacy. In a mouse total skin defect model, this hydrogel was loaded with mouse epidermal growth factor (mEGF) to accelerate wound repair and promote the regeneration of fresh tissue.

### 3.2. Heart

**Figure 7:**
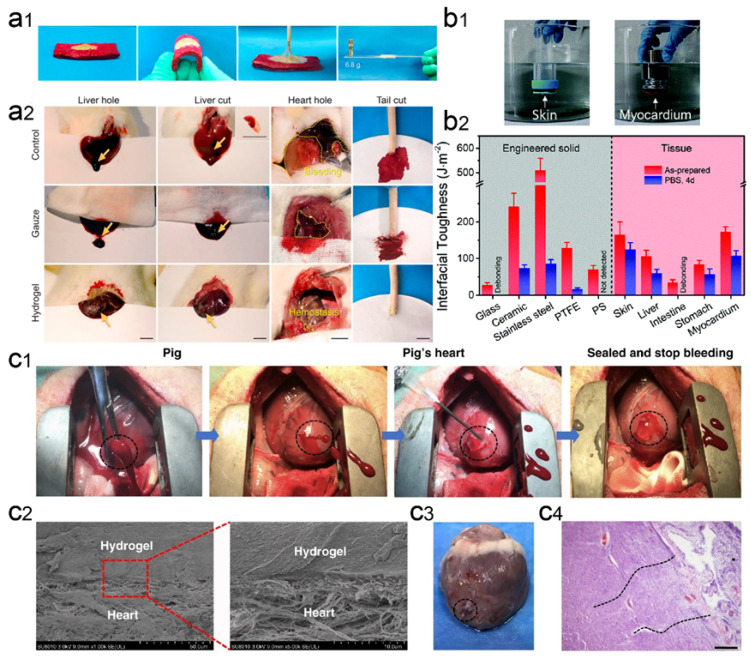
Wet-adhesive hemostatic hydrogel in heart wounds. (**a**) Adhesion and hemostatic properties of CS/TA/SF hydrogels. Figure modified from [96] with permission. (**a1**) The photos show that the hydrogel has strong adhesion to wet pigskin and glass. (**a2**) Hemostatic images of various untreated wounds covered with gauze or hydrogel. (**b**) Strong adhesion properties of PCT hydrogels. Figure modified from [97] with permission. (**b1**) Optical image of direct adhesion of PCT hydrogel between high-density polyethylene substrate and porcine skin or myocardium in PBS environment. (**b2**) Interfacial toughness of PCT hydrogels with different substrate surfaces before and after 4 days of immersion in PBS. (**c**) GelMA/HA-NB hydrogel adhesion and hemostatic properties. Figure modified from [98] with permission. (**c1**) Optical image of rapid hemostatic closure after a heart puncture wound. Blood exudation completely stopped within 10 seconds. (**c2**) Scanning electron micrographs of the interface between a porcine heart puncture wound and hydrogel. (**c3**) Autopsy images of the heart were performed after two weeks of postoperative recovery. (**c4**) Tissue-stained image of the interface between the heart tissue and the matrix gel of a pig heart 2 weeks after postoperative recovery.

Cardiac bleeding can occur as a result of trauma, such as injuries from accidents and wounds to the heart. Certain diseases can also lead to the rupture of blood vessels in the heart. Rapid and strong adhesion to wetted tissue walls and surfaces, high mechanical strength, and good biocompatibility to promote tissue regeneration are central and necessary for rapid hemostasis of cardiac arterial dissection. It has been shown that tannin (TA) contains a large number of benzene rings, which can make hydrogels very sticky in humid environments. When TA interacts with gelatin, CS, filamentous fibrin (SF), and Pluronic F127 (PEO_99_-PPO_65_-PEO_99_), low-swelling hydrogels with good mechanical and wet adhesive capacity were formed [96]. The obtained CS/TA/SF hydrogels showed less bleeding and shorter hemostasis time in various arterial and visceral bleeding models compared to previously reported materials (Figure 7a). Later in another study, Liang et al. created a physicochemical double network cross-linked hydrogel (PCT) using acrylic acid, CS, and TA as the main components [97]. The hydrogels have many active sites on their surfaces, allowing fast, strong and repetitive adhesion to artificial solids and biological tissues (Figure 7b). Due to its amide covalent bond, the hydrogel can act for a longer period in tissue regeneration, and the resulting hydrogel-tissue adhesion interface has strong adhesion even after one month of immersion in a physiological environment. Due to its platelet adhesion and high bursting pressure qualities, this hydrogel can be used for good hemostatic properties at sites of heavy bleeding such as the heart.

Bionic strategies are also effective in wet adhesion hemostasis. It has been shown that the cationic polysaccharide intercellular adhesion contained in staphylococcal biofilms plays a key role in surface adhesion to wet and moving surfaces [99]. Inspired by the strong adhesion mechanisms of biofilms and mussels, Han et al. reported a novel dual bionic adhesion hydrogel (DBAH), which is based on CS-grafted methacrylate (CS-MA), DA, and N-hydroxymethylacrylamide (NMA) [46]. When DBAH was contacted with water, hydrophobic residues (-CH_3_) rapidly generated cohesive forces that self-repel water molecules from the substrate surface. At this time, the catechol group of DA and the cationic free amine group NH_3_^+^ of CS-MA are exposed outward to promote sufficient contact with the adherent matrix for rapid and firm wet tissue adhesion, resulting in excellent hemostasis of DBAH even in the wet and active rabbit heart environment. In another study, Hong et al. designed a photo-responsive biological tissue adhesive that mimics the ECM composition. It consisted of GelMA, N-(2-aminoethyl)-4-(4-(hydroxymethyl)-2-methoxy-5-nitroso)butyramide modified glycosaminoglycan hyaluronic acid (HA-NB), and the polymerization initiator lithium phenyl-2,4,6-trimethylbenzoyl phosphate. Under UV light, this biomolecule-based hydrogel matrix rapidly gelled, adhered, and sealed the bleeding arteries and heart walls. This hydrogel can withstand a blood pressure of up to 290 mm Hg compared to most clinical situations (systolic blood pressure of 60–160 mm Hg). Notably, the hydrogel inhibited high-pressure hemorrhage from a 6-mm diameter cardiac perforation in the porcine heart and a 4- to 5-mm long incisional lesion in the porcine carotid artery [98] (Figure 7c).

### 3.3. Liver

**Figure 8:**
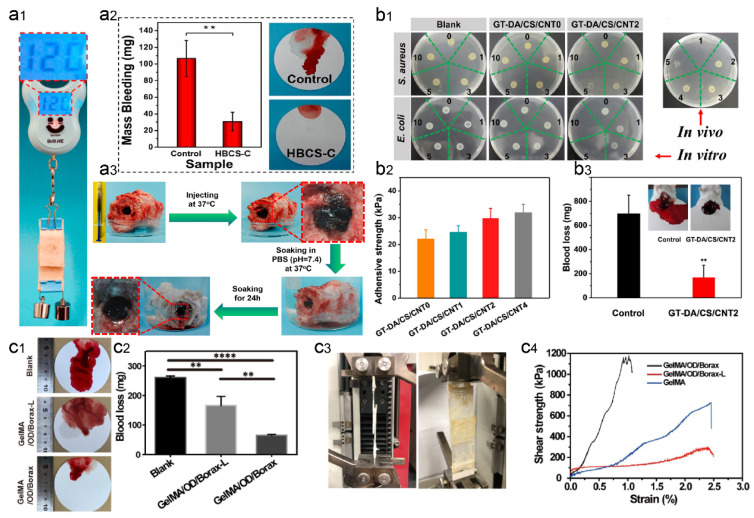
Wet-adhesive hemostatic hydrogel in liver wounds. (**a**) Tissue adhesion and hemostatic effect of HBCS-C hydrogel. Reprinted (adapted) with permission from [100]. Copyright {2020} American Chemical Society. (**a1**) The images show good adhesion of HBCS-C hydrogel on porcine skin (adhesion area of 20 mm × 20 mm and heavy load of 120 g). (**a2**) Total blood loss from liver wounds in hydrogel-treated and untreated rats. (**a3**) Photographs showing the wet bioadhesive behavior and stability of HBCS-C hydrogels in an aqueous environment at 37 °C. (**b**) Antimicrobial, adhesion, and hemostatic properties of GT-DA/CS/CNT hydrogel. Figure modified from [102] with permission. (**b1**) In vitro antibacterial activity of hydrogels induced by NIR illumination, 0, 1, 3, 5, and 10 represent different irradiation times (min). (**b2**) Adhesion strength of the hydrogels after 1 h in the air before testing. (**b3**) Hemostatic ability of GT-DA/CS/CNT hydrogels. (**c**) Adhesion and hemostatic properties of GelMA/oxidized dextran/Borax hydrogels. Figure modified from [101] with permission. (**c1**) Photographs of liver blood loss after different treatments. (**c2**) Blood loss during hemostasis of the liver. (**c3**) Pictures of the modified test method used for the overlapping shear test. (**c4**) Strain–stress curves for the overlapping shear test. (** *p* < 0.01, **** *p* < 0.001).

To achieve rapid and effective hemostasis of liver bleeding, Shou et al. designed a catechol-hydroxybutyl CS (HBCS-C) hydrogel by attaching catechol and hydroxybutyl molecules to a CS backbone. This multifunctional HBCS-C hydrogel showed thermal sensitivity, injectability, tissue adhesion, and biocompatibility [100]. The multiple interactions between catechol hydroxyl/amino groups and tissues allow the biocompatible hydrogel to adhere firmly to the tissue surface. The hydrogel effectively blocked the bleeding in a rat liver hemorrhage model by adhering firmly to the bleeding tissue within 30 seconds (Figure 8a). In another work, Chen et al. created an in situ-generated hemostatic hydrogel (GelMA/oxidized dextran/Borax) for incompressible visceral wound hemostasis and anti-inflammatory applications [101]. The abundant adjacent hydroxyl groups of dextran can be oxidized to aldehyde groups by sodium periodate, which can be further bound to the amino groups of histones by Schiff base reaction, giving dextran good tissue adhesion ability. In addition, sodium tetraborate produces dynamic borate ester linkages when combined with oxidized dextran. Due to the three-layer network structure, the hydrogel exhibits good hemostatic capacity and can withstand high blood pressure exceeding 165 mm Hg, which is higher than the systolic blood pressure threshold for healthy adults (i.e., 120 mm Hg) (Figure 8c).

Various multifunctional injectable hydrogels for hemostasis have been successfully developed, however, these strategies ignore issues such as the ease of removal of these sealants on injured livers and the occurrence of secondary injuries. To address this issue, Bu et al. created a rapidly formed aminolysis tetrapolyethylene glycol (Tetra-PEG) hydrogel sealant which has good mechanical strength and tissue adhesion [103]. The cyclized succinyl ester moiety gave the sealant adjustable solubility and fast decomposition properties. The hydrogel showed high hemostatic activity even in the presence of anticoagulation and exhibited remarkable biocompatibility and utility. The drug loading will allow the hydrogel to accelerate wound healing by enhancing the antibacterial and anti-inflammatory effects and hemostatic effects. Liang et al. used gelatin graft-dopamine (GT-DA) and polydopamine-coated carbon nanotubes (CNT-PDA) to design antibacterial, adhesive, antioxidant, and conductive GT-DA/CS/CNT composite hydrogels by oxidative coupling of catechol moieties using an H_2_O_2_/HRP catalytic reaction [102]. Then, the antibiotic doxycycline was added to the hydrogel. Together with the photothermal effect of CNT-PDA, the hydrogels gained good antimicrobial action and showed well in vitro and in vivo antimicrobial activity against different microorganisms. In a mouse model of liver hemorrhage, mice in the untreated group drained approximately 700 mg of blood from the liver, while mice in the gelatin-DA/CS/CNT hydrogel group shed only 170 mg of blood (Figure 8b). Furthermore, the GT-DA/CS/CNT hydrogels also exhibited good tissue regeneration capacity, as indicated by the collagen deposition, histomorphometric analysis, and immunofluorescence staining for transforming growth factor (TGF) and a cluster of differentiation 31 (CD31). In a study, He et al. developed a conductive self-healing hydrogel for hemostasis using N-carboxyethyl chitosan (CECS), PF127, and CNT as the main ingredients, and further loaded with moxifloxacin hydrochloride [104]. A mouse liver injury model, a mouse liver incision model, and a mouse tail amputation model were used to evaluate the hemostatic ability of the CECS/PF127/CNT hydrogel. It was found that the hydrogel could adhere to the wound site and form the hydrogel within a short period (75 s), which acted as a stable physical barrier and prevented wound bleeding. In addition, the negative charge of CECS triggered an intrinsic coagulation pathway, which ultimately formed a stable platelet plug during hemostasis. Therefore, the blood loss in the CECS/PF127/CNT hydrogel group was significantly less than that in the control group. 

### 3.4. Other Applications

Many gastric diseases can cause gastric bleeding with gastroscopy as well as with surgical treatment [105]. To solve this problem, He et al. prepared injectable pH-responsive self-healing adhesive hydrogels based on acryloyl-6-aminohexanoic acid (AA) and AA-gN-hydroxysuccinimide (AA-NHS) with efficient self-healing ability, hemostatic properties, and good biocompatibility. With the introduction of AA-NHS as a micro-crosslinker, the hydrogels exhibited enhanced adhesive strength. An in vivo model of porcine gastric bleeding showed that the hydrogel displayed good hemostatic properties by stopping acute arterial bleeding and preventing the bleeding. Gastric wound models showed that hydrogels exhibited excellent therapeutic effects, with type I collagen deposition, α-SMA expression, and angiogenesis significantly promoting wound healing [106]. In another study, Cheng et al. created an oral keratin hydrogel that adheres specifically to ulcers and accelerates ulcer healing [107]. In ethanol-treated rats, approximately 50% of the ulcer-adherent keratin hydrogel was able to remain in the stomach for 12 hours, however, only approximately 18% was left in healthy rats during the same period. In addition, keratin hydrogels prevented epithelial cell damage by gastric acid, reduced inflammation, and accelerated epithelial reconstitution of ethanol-induced gastric ulcers. 

Intestinal bleeding is a bleeding disorder that may be caused by inflammation, mechanical injury, vascular lesions, or tumors. Recently, Cui et al. designed a high-strength instant self-adhesive organic-inorganic hybrid (OIH) hydrogel. This OIH hydrogel was composed of N-acryloyl 2-glycine (ACG), a biocompatible glycine derivative vinyl monomer, and the naturally occurring mineral hydroxyapatite (HAp) [108]. The hydrogen bonding of the poly(N-acryloyl 2-glycine) (PACG) side chains, the carboxyl-calcium ion crosslinking, and the PACG chain-HAp physical interactions contribute to the high mechanical properties of the hydrogel. Importantly, this OIH hydrogel exhibited strong adhesion to different substrates, which could be attributed to the synergistic interaction of carboxyl groups and the enhanced contact of PACG chains with the adherent surface.

## 4. Summary and Outlook

Different substances have been discovered to stop bleeding, however, their effectiveness depends on the type of bleeding and the location of the wound. Although many effective hemostatic bandages, gauze, foams, sprays, adhesives, gels, powders, granules, tourniquets, and tampons have been discovered for externally visible and accessible, and compressible wounds, these uncontrollable and incompressible bleedings remain a challenging problem in treating the internal arterial visceral bleeding and massive surgical blood loss. To stop blood loss, hemostatic hydrogels must firmly adhere to specific tissues under high fluid pressure, moisture, and dynamics while maintaining their good mechanical strength. In recent years, there has been a dramatic increase in the availability of multifunctional polymeric hydrogels to fill the current needs in wound management. In this review, we discussed recent advances in wet-adhesive hydrogel systems as hemostatic agents, sealants, or adhesives, focusing on elucidating the mechanisms of hemostasis, the various factors affecting wet-environment adhesion, and the application of wet-adhesive hemostatic hydrogels in various complex physiological settings such as skin, cardiac and liver wounds, and other mucosal bleeds. Some of the polymer precursors were modified with catechol as well as other groups to provide strong adhesion and good hemostatic properties. These developed hydrogels are not only highly biocompatible but also can effectively exert antiseptic and anti-inflammatory effects when antimicrobial substances are added, thereby promoting wound healing. Moreover, these hydrogels can withstand high blood pressure and have shown advantages over some existing commercial products.

Despite improvements in recent years in research on wet-adhesive hydrogel systems for hemostasis and wound healing, there are still many hurdles that need to be addressed before these hydrogel technologies can be used in the clinic. First, few hydrogels have been able to stop bleeding quickly and effectively in the presence of excess water and heavy bleeding from active tissue. Additional researches are necessary to develop effective injectable hydrogels that can effectively and accurately stop moist, smooth, and dynamic internal tissue and organ bleeding. Second, high-quality raw materials used to prepare wet-adhesive hydrogels should be exploited. Although hemostatic hydrogels prepared by mussel-inspired chemical methods have shown good adhesion and hemostatic properties, substances such as DA are expensive as raw materials for the preparation of these hydrogels, and the potential neurological side effects of DA would limit their mass production and commercialization [109]. There are also low-cost and safe substances considered alternatives to DA, like plant-derived polyphenolic compounds such as TAs containing catechol/pyrogallol molecules. Nevertheless, it should be noted that additional stimuli are required to stimulate the formation of polymer-TA hydrogels [110,111]. Moreover, the incorporation of artificial nanoenzymes with antioxidant properties to fabricate a multifunctional hemostatic hydrogel may also facilitate their translation [112]. Therefore, the development of safe raw materials for viscous hydrogels, as well as the development of new strategies to achieve rapid and effective hemostasis of hydrogels in humid environments, remain to be explored in the future, and it is hoped that this review will inspire future development in this field. 

## Data Availability

Not applicable.
